# Meager Diet of Porto Rican Peasantry

**Published:** 1899-03

**Authors:** 


MEAGER DIET OF PORTO RICAN PEASANTRY.—Dr. L. Amadeo, a physician resident in Porto Rico, has made a careful report on the mental and physical condition of his fellow-islanders. He was well qualified for undertaking the investigation, of which the summarized results were published in the New York journals, for he had both studied and practiced medicine in this country, and, being a Porto Rican by blood and birth, was not likely to see in his people defects that do not exist. What he did see was the effect of insufficient nutrition, extending through the whole working class, i. e., through the whole class, which, thanks to Spanish tariffs, was forced to live on roots and fruits. He implores his new rulers to avoid the murderous policy of their predecessors, and to put within the reach of the toiling thousands in Porto Rico the very food which so horrifies and disgusts the vegetarian monomaniacs. Meat, he says, is the remedy for wasted muscles and inactive brains. This is science; it is also common sense, but it will raise a howl of anger from the unfortunate people to whom the prohibition of good things seems to be the alternative to its abuse.—*New York Times.*

